# Supplementary material for: scellop: a scalable redesign of cell population plots for single-cell data
Source: Bioinform Adv. 2026 Mar 21;6(1):vbag083. doi: 10.1093/bioadv/vbag083 (PMC13050608; doi:10.1093/bioadv/vbag083)
Supplement: vbag083_Supplementary_Data [file vbag083_supplementary_data.pdf]

Supplementary Materials for Smits et al. (2026). scellop: a scalable redesign of cell population plots for single-cell data. Bioinformatics Advances, vbag083. https://doi.org/10.1093/bioadv/vbag083

Supplementary Materials

Unique Cell Type Calculation

For calculating the average number of cell types, we used 162 datasets from the Human BioMolecular Atlas Program (HuBMAP). We retrieved the cell types with *scellop*'s data loaders, and calculated the number of unique cell types per dataset. We used the following datasets, identified by their HuBMAP ID:

HBM964.BVWP.737	*HBM675.VBDH.688*	*HBM846.KVCF.674*	*HBM266.FTJN.632*	*HBM546.RNHX.756*	*HBM528.KNCB.488*
HBM729.TVMN.534	*HBM734.LFLC.264*	*HBM232.MBNR.586*	*HBM965.PSNC.855*	*HBM482.MCCP.264*	*HBM796.PCWD.863*
HBM752.KZCK.589	*HBM373.HCFG.722*	*HBM467.RQDN.922*	*HBM626.PHCW.834*	*HBM528.DMSV.294*	*HBM324.XBMF.465*
HBM628.QKGB.497	*HBM462.XQCR.933*	*HBM456.CGDP.395*	*HBM976.MRWH.263*	*HBM979.VMDC.365*	*HBM647.QDBG.936*
HBM938.GBST.823	*HBM936.MHTZ.834*	*HBM444.PWKX.639*	*HBM674.FLVW.576*	*HBM299.VDWT.444*	*HBM456.XDCK.572*
HBM892.JLFV.844	*HBM437.LCSH.956*	*HBM775.CMGG.464*	*HBM937.TWRN.355*	*HBM334.DWWF.436*	*HBM736.MNMD.453*
HBM532.KKRC.477	*HBM539.GJNB.784*	*HBM759.CHJW.244*	*HBM247.HLXR.494*	*HBM969.PBMH.689*	*HBM699.XBTD.684*
HBM735.FSBZ.626	*HBM679.RLJH.432*	*HBM823.CNRW.484*	*HBM597.PBJW.593*	*HBM253.ZBGF.863*	*HBM563.FFQJ.764*
HBM222.VQSW.335	*HBM847.MDSJ.826*	*HBM547.SJSK.268*	*HBM883.PHQS.523*	*HBM629.GSHG.922*	*HBM264.MJCH.639*
HBM634.JHVB.286	*HBM363.NTWP.766*	*HBM439.BQLR.867*	*HBM928.THDD.545*	*HBM874.JPGB.398*	*HBM368.JCBG.263*
HBM634.ZSHF.736	*HBM766.NZWP.682*	*HBM648.DKQK.874*	*HBM842.DDTX.473*	*HBM476.ZLDJ.925*	*HBM363.FVKP.935*
HBM595.LBXP.486	*HBM793.JDRF.289*	*HBM633.LLDZ.679*	*HBM949.PLLF.787*	*HBM694.NXCN.368*	*HBM938.WTSR.492*
HBM398.BLRW.228	*HBM976.LDTR.982*	*HBM297.FDTX.382*	*HBM459.KCST.593*	*HBM779.FQMX.497*	*HBM986.KFWG.239*
HBM292.GSZL.269	*HBM827.MJMM.447*	*HBM522.VFGB.335*	*HBM248.HPXX.584*	*HBM445.HBRQ.488*	*HBM782.HVML.355*
HBM845.SFMK.942	*HBM547.TFRR.794*	*HBM787.XCSX.733*	*HBM975.MVDK.648*	*HBM892.CCDZ.345*	*HBM834.SLQN.292*
HBM593.CLXN.573	*HBM269.GDLH.894*	*HBM485.VKSZ.779*	*HBM294.XZLM.256*	*HBM933.JFFT.692*	*HBM356.MDPN.792*
HBM757.KLKW.524	*HBM785.XFTT.663*	*HBM425.GDJT.648*	*HBM522.QXVG.468*	*HBM522.FTFK.487*	*HBM449.QGGL.994*
HBM745.FJML.722	*HBM688.RPFC.258*	*HBM894.DMKD.525*	*HBM573.JGLL.575*	*HBM478.VWXX.362*	*HBM573.JMXM.823*
HBM762.RPDR.282	*HBM727.DWPV.852*	*HBM468.SSXX.967*	*HBM887.DDJL.589*	*HBM589.THRM.428*	*HBM859.LTWK.468*
HBM874.PWHS.622	*HBM858.MFWR.937*	*HBM982.DSNZ.722*	*HBM972.LBGS.258*	*HBM975.WQQQ.853*	*HBM832.WTNH.257*
HBM967.LPHM.957	*HBM444.DXLZ.643*	*HBM726.NFVH.245*	*HBM362.DZVK.533*	*HBM975.JGXC.665*	*HBM883.DKXZ.574*
HBM864.CWHJ.963	*HBM929.VSJQ.633*	*HBM456.GRCM.369*	*HBM482.DKQF.747*	*HBM582.CXXZ.438*	*HBM339.BGVK.388*
HBM932.ZMRS.894	*HBM373.SCNK.776*	*HBM625.BCND.537*	*HBM343.XKRX.239*	*HBM735.NMFW.852*	*HBM293.QVMW.765*
HBM487.MCTL.254	*HBM873.PZTG.367*	*HBM529.KHGN.262*	*HBM265.FQWZ.384*	*HBM675.RVGB.258*	*HBM798.BBXD.333*
HBM269.XWMK.444	*HBM384.FLVW.984*	*HBM778.JJDB.736*	*HBM326.WHVS.274*	*HBM948.GXMD.986*	*HBM578.BDBP.672*
HBM673.GSSW.364	*HBM492.CQJD.323*	*HBM473.RKXT.944*	*HBM982.THGM.772*	*HBM342.CMHT.948*	*HBM599.GNJN.777*
HBM957.TXXZ.387	*HBM385.LQVK.975*	*HBM927.DZCV.762*	*HBM537.MVDQ.934*	*HBM236.JPVT.769*	*HBM852.VXGN.375*

User Study

We conducted a user study on cell population plots as part of the HuBMAP Data Portal. Fourteen participants participated in 30 minute semi-structured interviews focused on cell population visualizations. Participants were recruited for their domain expertise in single-cell and spatial biology, with 12 self-identifying as experimental biologists, 5 computational biologists, 5 educators, 1 clinician, and 12 data contributors to HuBMAP. Their expertise covered 11 different organs. Most participants specialized in multi-omics approaches and bulk, single-cell and spatial transcriptomics as well as (multiplex) fluorescent imaging, while some focussed on mass spectrometry and (3D) imaging.

Users were first asked about their desired visualizations and interactions for exploring cell types prior to being shown any example visualization. They were then asked to examine the cell population plot available at https://hubmapconsortium.github.io/tissue-bar-graphs/ (with 'Kidney HuBMAP Portal (107 datasets)' as source, and default sorts and groupings), also shown in Figure S1.

Users highlighted the importance of comparison of datasets and cell types. Users wanted to find relations between cell type distributions and metadata, specifically within demographic groups and with different cell types. Users also wanted to see the total number of cells, the most frequent cell type, and find the cell types with highest and lowest cell counts between datasets.

The desired interactions raised by multiple users were: Normalization Option [N=10], Group by Cell Type Hierarchy [N=9], Overview with Detail [N=9], Ability to manipulate visualization (e.g. selecting substacks, filtering, etc.) [N=8], Additional Context [N=5], Group samples by desired feature [N=5], Filter by cell types [N=4], Filter by data types [N=4], Filter by donor metadata [N=4], Filter by anatomical structures [N=3], Filter by biomarkers [N=2]. Issues raised by multiple users were: Color Scheme [N=6].

Additional points raised were that there were too many cells, that it was hard to see the big picture, that cell types can vary by granularity. Additional interactions raised by individuals were cell type sorting by prevalence, seeing all samples at once, and downloading the graph as an image. One user specified that they normally looked at at least 30 datasets at one time.

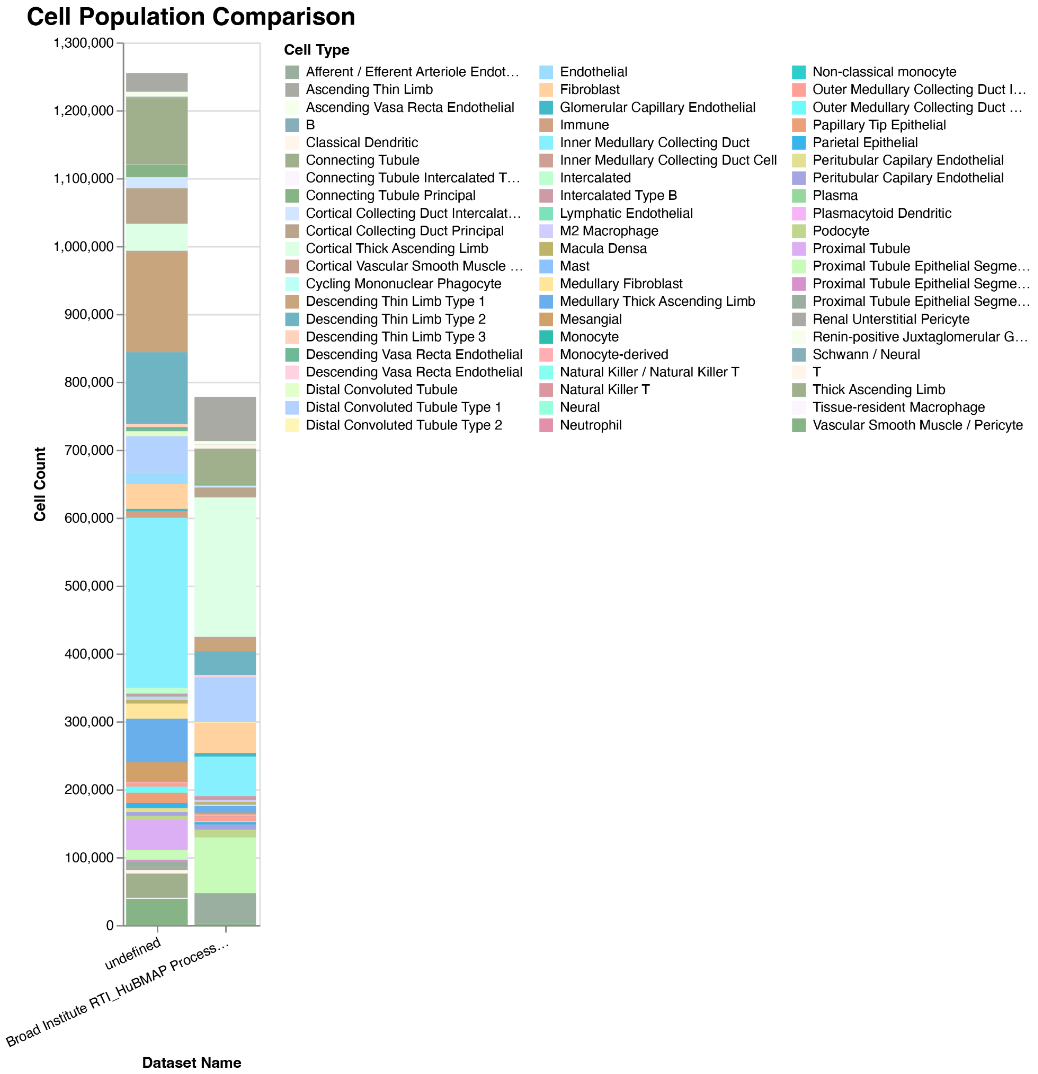

Figure S1. Cell Population Comparison used in HuBMAP Data Portal user study, with 63 cell types and two groups.

Performance

To demonstrate *scellop*'s utility on single-cell datasets, we include performance metrics for various data sizes (Table S1). We used real dataset and simulated datasets of various sizes (Table S1). We used the *vitest* (https://vitest.dev/) framework to test the performance of different operations of *scellop* on these datasets. To determine the time per operation, each operation was run several times. First, a number of warmup iterations was run to stabilize the node compiler. Operations were then run for 500ms, with a minimum of 10 times for operations lasting longer than 50ms. We denote the mean operation speed per operation (Table S2).

Table S1. Datasets used for determining performance metrics.

Dataset	Type	Dimensions	Entries	Non-Zero Cells Density (%)
tiny	synthetic	10×10	100	82
small	synthetic	50×50	2500	59.6
hubmap-lung	real-world	45×71	3195	44.9
hubmap-kidney	real-world	108×48	5184	73.1
medium	synthetic	100×100	10000	41.5
extraWide	synthetic	20×1000	20000	25.1
extraTall	synthetic	1000×20	20000	25.4
wide	synthetic	50×500	25000	29.9
tall	synthetic	500×50	25000	30.4
large	synthetic	200×300	60000	30.1
hca-data	real-world	484×51	24684	48
huge	synthetic	500×500	250000	19.9

Table S2. *scellop* visualization performance metrics.

Dataset	DataMap creation		Render pipeline		Export pipeline	
	Mean (ms)	*SD (ms)*	*Mean (ms)*	*SD (ms)*	*Mean (ms)*	*SD (ms)*
tiny	0.0	0.1	0.0	0.3	0.0	0.0
small	0.2	0.1	0.6	1.2	0.8	0.3
hubmap-lung	0.9	0.5	0.8	1.0	1.5	0.4
hubmap-kidney	1.3	0.4	1.6	1.6	2.3	0.3
medium	0.8	0.4	2.8	2.4	3.5	0.6
extraWide	1.0	0.4	8.5	4.1	9.3	0.9
extraTall	1.0	0.4	7.2	2.6	8.7	1.2
wide	1.8	1.0	10.5	3.9	12.2	1.7
tall	1.8	0.9	10.1	2.3	11.5	1.1
large	4.7	1.4	24.8	6.1	25.0	1.8
hca-data	9.4	2.4	9.3	3.2	19.8	3.9
huge	19.4	3.2	127.8	20.2	128.8	4.7

Patterns in *scellop*

scellop allows for pattern detection in datasets, such as those from donors with different diseases (Figure S2).

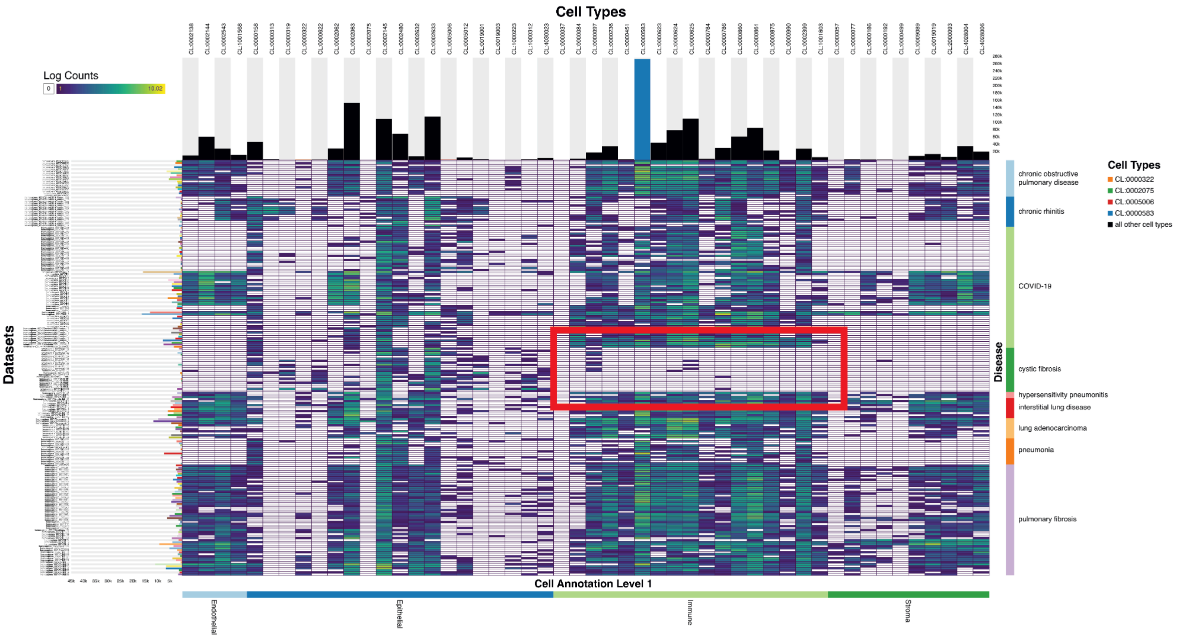

Figure S2. Human Lung Cell Atlas datasets in *scellop*. The area with immune cells for donors with cystic fibrosis is highlighted.
